# The association between prosocial behavior and subjective well-being among Chinese post-00s youth: the roles of meaning in life and prosocial motivation

**DOI:** 10.3389/fpsyg.2026.1887620

**Published:** 2026-07-29

**Authors:** Yunlong Yao, Hanxiao Zhang, Jinghao Feng

**Affiliations:** 1 School of Social Development, Hunan Women’s University, Changsha, China; 2 School of Psychology, Yunnan Normal University, Kunming, Yunnan, China; 3 Department of Sociology & Psychology, School of Public Administration, Sichuan University, Chengdu, China

**Keywords:** Chinese post-00s generation, meaning in life, prosocial behavior, prosocial motivation, subjective well-being

## Abstract

**Introduction:**

The “Post-00s” generation in China, shaped by rapid social transition, increasingly prioritizes individualistic goals, which often reduces their motivation toward prosocial behavior. This individualistic tendency, however, conflicts with the collectivist cultural expectations in China that strongly endorse prosocial engagement.

**Methods:**

Therefore, the current study investigated whether more prosocial behavior was associated with subjective well-being (SWB) among a sample of Chinese university students born after 2000 (*N* = 1,084), specifically examining the parallel mediating roles of the search for meaning and the presence of meaning, as well as the moderating influence of prosocial motivation.

**Results:**

Results from a moderated parallel mediation model revealed that prosocial behavior was positively associated with SWB through both dimensions of meaning in life. Crucially, the strength of prosocial motivation significantly moderated this indirect pathway. Under high prosocial motivation, the positive associations between prosocial behavior, meaning in life, and SWB were robustly stronger. Conversely, when prosocial motivation was low, the indirect pathway via the search for meaning became non-significant, and the overall positive association with SWB was substantially weakened.

**Discussion:**

This study highlights the importance of integrating motivational strength into prosociality research and offers culturally relevant insights into well-being processes among contemporary Chinese youth.

## Introduction

Subjective well-being (SWB) refers to individuals’ cognitive and affective evaluations of their lives, which is essential for better mental and physical health, stronger social relationships, and greater resilience in the face of life’s challenges (Diener et al., 2018). While a large body of research has shown that prosocial behavior, acting to benefit others, is an important driver of SWB (Aknin et al., 2013; Curry et al., 2018; Dunn et al., 2008; Hui et al., 2020), this common belief that “doing good” always leads to “feeling good” has been challenged by inconsistent findings in recent studies (Li et al., 2023). This inconsistency could be explained by the Self-Determination Theory, which suggested that the pursuit of subjective well-being is not only rooted in prosocial behavior but also motivation (SDT; Ryan and Deci, 2017). As China’s “Post-00s” generation navigates a complex cultural landscape characterized by both deeply rooted collectivist norms and a rapid rise in modern individualistic values, the strength of internal prosocial motivations has become increasingly intricate (Dong et al., 2025; Yan, 2010). Growing up in a highly competitive social and academic environment, these individuals frequently face a psychological tension between societal expectations of altruism and personal demands for resource optimization (Sun et al., 2011). However, how the strength of this internal drive moderates the relationship between prosocial behavior and SWB requires further investigation. Therefore, the present study focuses on a sample of Chinese “Post-00s” Generation to explore the interactive and underlying pathways of prosocial behavior and motivation on subjective well-being.

The robust association between prosociality and subjective well-being is often attributed to the “warm glow” effect, where the act of giving triggers immediate emotional rewards (Bianchi et al., 2023; Chen, 2023; Dunn et al., 2008). Meta-analytic evidence confirms that engaging in prosocial behavior generally correlates with higher subjective well-being across diverse cultures and age groups (Hui et al., 2020). Specifically, global data suggests that prosocial spending consistently predicts higher subjective well-being, even after controlling for individuals’ income levels (Aknin et al., 2013). Even during the recent global health crisis (COVID-19), research highlighted that frequent prosocial behavior served as a buffer against psychological distress, fostering greater resilience (Chong et al., 2021; Lin et al., 2025). However, this generalized optimism masks significant empirical and methodological limitations. A critical one in existing literature is the frequent conflation of prosocial attitudes with actual prosocial behaviors. Many studies rely on dispositional scales that measure an individual’s general willingness or “prosocial tendency” (e.g., “I am willing to help others”) rather than the actual frequency of prosocial behavior performed (Baumsteiger and Siegel, 2019; Furnell et al., 2025). Such measures are highly susceptible to social desirability bias, where participants over-report altruistic intentions to align with social norms (Awan et al., 2020). As expressing an attitude requires negligible personal cost, these measurements often fail to capture the “resource drain” inherent in real-world helping. Consequently, the positive link between prosociality and subjective well-being may be overstated in studies that measure intent rather than action, leading to a fragmented understanding of the conditions under which “doing good” is less strongly associated with emotional benefits.

Moreover, recent longitudinal studies and field experiments suggest that the emotional benefits of helping are not universal; in some contexts, prosocial efforts show no measurable association with well-being, or even correlate with “prosocial burnout” and increased psychological distress (Jaeger and van Vugt, 2022; Li et al., 2023). For example, experimental evidence has demonstrated that when helping is perceived as a “compulsory citizenship behavior” or driven by high social pressure, it can lead to emotional exhaustion and reduced job satisfaction rather than a “warm glow” (Liang et al., 2022). Similarly, research focusing on high-cost or obligatory support, such as caregiving or intensive emotional labor, indicates that excessive prosocial engagement can drain personal resources, leading to a decline in affective well-being (Krämer and Bleidorn, 2024). Even in daily life contexts, longitudinal data reveal that the positive link between helping and happiness disappears when individuals lack the necessary psychological resources or feel their autonomy is compromised (Kesenheimer et al., 2023). These mixed findings suggest that prosocial behavior is not a monolithic correlate of well-being. Instead, the existing literature reveals a critical tension: while the frequency of prosocial behavior is often measured, the strength of the underlying experience is frequently overlooked. This oversight has led to a fragmented understanding of the conditions under which “doing good” becomes an emotional burden rather than a benefit.

To resolve this fragmentation, it is essential to shift the focus from the surface-level frequency of actions to the strength of the prosocial motivation that initiates them. Self-Determination Theory (SDT) provides a robust framework for this relationship (Ryan and Deci, 2017). In the context of prosociality, prosocial motivation reflects the intensity of an individual’s desire to benefit others (Grant and Sumanth, 2009). According to SDT, the emotional rewards of prosociality are not guaranteed by the behavior itself; rather, they may depend heavily on how strongly the individual actually wants to help (Weinstein and Ryan, 2010). When frequent prosocial behavior is accompanied by high prosocial motivation it represents an intense personal drive to contribute to others, which is associated with a sense of fulfillment (Weinstein and Ryan, 2010). Conversely, when prosocial behavior is performed by individuals with low prosocial motivation, meaning the genuine desire to help is weak, the act may lack personal significance (Lin et al., 2019). This indicates that prosocial behavior does not inherently boost well-being; instead, its positive impact is conditional, depending entirely on the strength of the individual’s internal drive to help.

Building on this logic, meaning in life (MIL) represents the vital psychological variable that translates the quality of prosocial engagement into long-term subjective well-being (Wang et al., 2024). To capture this process, it is necessary to examine MIL through its two distinct dimensions: the presence of meaning and the search for meaning (Steger et al., 2006).

Regarding the presence of meaning, prosocial behavior acts as a natural “meaning-making” activity. It allows individuals to transcend their own needs and contribute to the welfare of others, which fosters a direct sense of being useful and valued in the world (Van Tongeren et al., 2016). This fulfills the human need for significance, purpose, and coherence (Martela and Steger, 2016). However, according to meaning-making frameworks (Steger et al., 2006), meaning is not merely a static endpoint but a dynamic, ongoing process. Therefore, prosociality also triggers a search for meaning. For university students navigating the transition to adulthood, engaging in prosocial behavior allows young adults to move beyond self-centered concerns and confront diverse social realities (Kim and Morgül, 2017). This exposure serves as a psychological catalyst for identity development. Rather than offering an immediate sense of purpose, prosociality stimulates deeper cognitive reflection regarding their societal roles and long-term life goals (Grant, 2007). Consequently, the practice of helping others fosters identity exploration, driving young adults to actively construct meaning and search for meaning in life.

**Figure 1:**
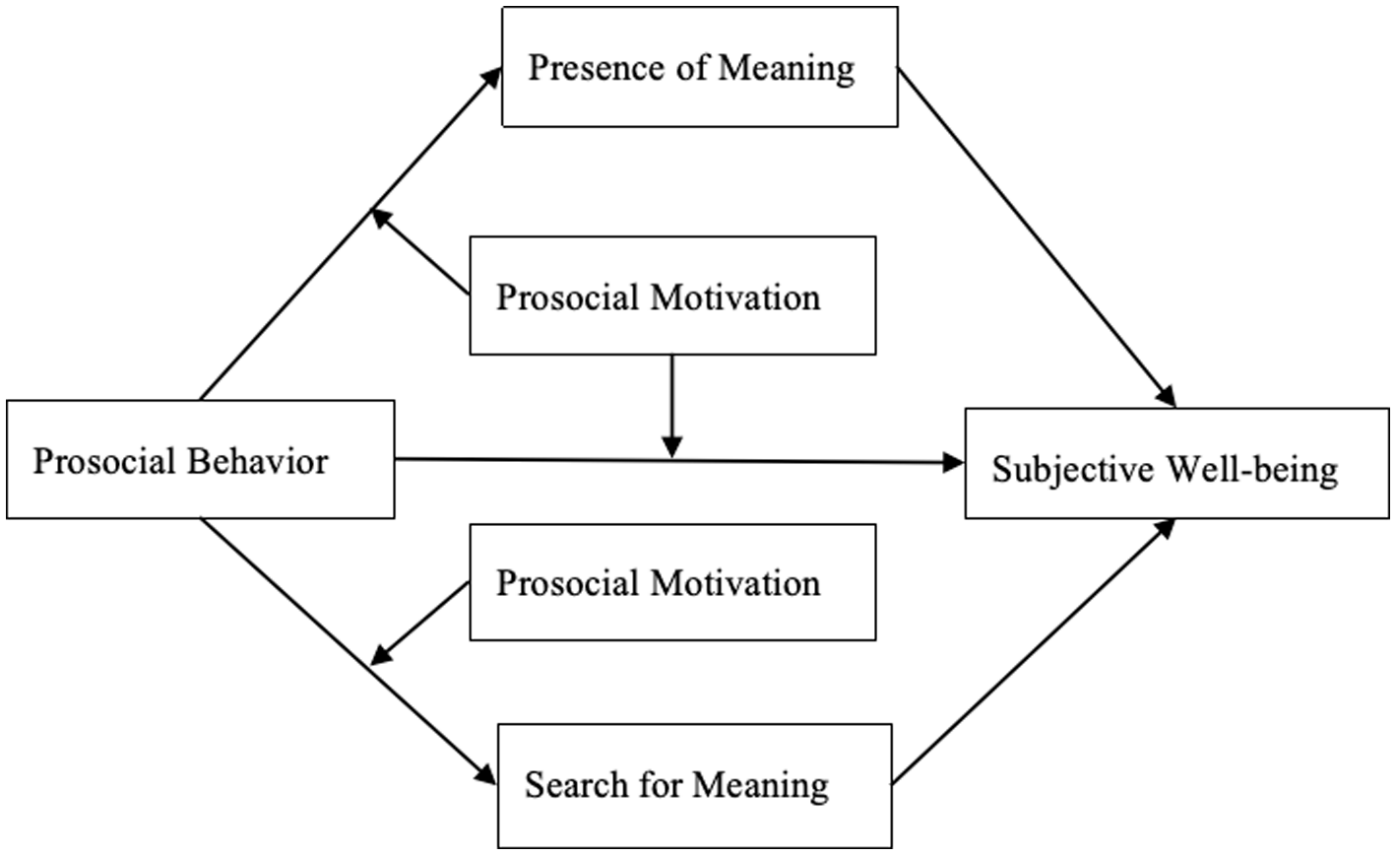
The hypothesized model.

Consequently, in the current study, we test our hypotheses within this population, measuring the self-reported frequency of participants’ prosocial behavior rather than their attitudes. We first propose that self-reported frequency of participants’ prosocial behavior is associated with higher level of subjective well-being (SWB) through the mediating role of meaning in life (MIL). Crucially, the strength of this association depends on the level of prosocial motivation. Specifically, we hypothesize that prosocial motivation moderates both the direct association of behavior on SWB and the indirect associations through the two dimensions of MIL: search for meaning and presence of meaning. When motivation is high, meaning the act is accompanied by a strong internal desire to help, prosocial behavior is expected to be strongly linked to greater meaning in life and higher subjective well-being. In contrast, when motivation is low, these positive relationships are weakened or lost. Figure 1 illustrates the theoretical model.

## Method

### Participants

This study employed convenience sampling to recruit participants from four universities in a city within Yunnan Province, China. The sample included students ranging from freshmen to the graduate level. Data collection was conducted using a hybrid offline recruitment and digital administration approach. Research assistants physically visited university classrooms and campus common areas to invite student participation. Participants accessed the questionnaire by scanning a unique QR code or following a secure link provided by the researchers, completing the measures on their personal mobile devices via an online survey platform. Of the 1,188 questionnaires distributed, 104 were excluded due to invalidity: 48 participants failed the embedded attention checks (lie scales), and 56 exhibited rapid responding (defined as a response latency of less than 2 s per item). This screening resulted in a final analytical sample of 1,084 valid responses, representing a valid response rate of 91.2%. Participants’ ages ranged from 17 to 24 years, with a mean age of 20.15 years (SD = 1.82). The demographic characteristics of the sample are presented in Table 1.

**Table 1:** Descriptive characteristic.

Variables	Category	*N*	Percentage (%)
Sex	Male	408	37.6%
	Female	676	62.4%
Residence	Urban	410	37.8%
	Rural	674	62.2%
Grade level	Freshman	308	28.4
	Sophomore	384	35.4%
	Junior	191	17.6%
	Senior	91	8.4%
	Graduate level and above	110	10.1%
Political affiliation	CPC members	99	9.1%
	Probationary members	526	48.5%
	Non-CPC member	459	42.3%

This study was reviewed and approved by the Academic Review Committee of Yunnan Normal University (Approval Date: August 15, 2024). Note that the committee does not issue specific approval numbers for minimal-risk survey research. All procedures performed in this study involving human participants were in accordance with the ethical standards. Active online informed consent was obtained from all participants on the first page of the survey prior to their participation; participants were required to check a box indicating their consent before proceeding to the questionnaire. They were fully informed about the study’s purpose, their right to withdraw at any time, and data confidentiality. For university students aged 17, the requirement for parental or guardian informed consent was formally waived by the Academic Review Committee of Yunnan Normal University, given the minimal psychological risk of the anonymous online survey and their status as independent university students.

### Measures

#### Demographics

A self-developed demographic questionnaire was used to collect participants’ background information. This included sex (male or female), political affiliation (CPC member, probationary members, and non-CPC member), age, and grade level (categorized as freshman, sophomore, junior, senior, or graduate level and above). Additionally, participants reported their place of residence (urban or rural).

#### Self-reported frequency of prosocial behavior

Self-reported frequency of prosocial behavior was assessed using the Chinese version of the Self-Report Altruism Scale (SRA; Rushton et al., 1981; Su, 2014). The revised scale is unidimensional and consists of 19 items involving typical prosocial behaviors in daily life; for example, “I have given directions to a stranger.” Participants performed a self-evaluation on a 5-point Likert scale ranging from 1 (never) to 5 (always), where higher scores indicate a higher frequency of prosocial behavior. In the present study, the scale demonstrated high internal consistency, with a Cronbach’s alpha of 0.915.

#### Meaning in life

The Meaning in Life Questionnaire (MLQ; Steger et al., 2006), revised into Chinese by Liu and Gan (2010), was used to measure participants’ sense of meaning in life. The scale consists of 9 items divided into two subscales: Presence of Meaning (MLQ-P), containing five items (e.g., “I understand my life’s meaning”), and Search for Meaning (MLQ-S), containing four items (e.g., “I am looking for something that makes my life feel meaningful”). Item 2, “My life has no clear purpose,” is reverse-scored. Responses are rated on a 7-point Likert scale ranging from 1 (strongly disagree) to 7 (strongly agree), with higher scores indicating a higher level of meaning in life. In the present study, the Cronbach’s alpha coefficient for the total scale was 0.866, and the coefficients for the Search for Meaning and Presence of Meaning subscales were 0.869 and 0.891, respectively.

#### Prosocial motivation

Prosocial motivation was measured using the Prosocial Motivation Scale developed by Grant and Sumanth (2009). This scale, built upon the work of Grant (2008) and VandeWalle (1997), has demonstrated strong theoretical foundations and psychometric properties. It was translated and revised into Chinese by Min (2015) and has shown good reliability and validity in previous research (Li et al., 2018). The scale consists of five items (e.g., “I like to do things that benefit others”). Participants responded using a 5-point Likert scale, ranging from 1 (strongly disagree) to 5 (strongly agree). Total scores are calculated by summing the item scores, with no reverse-scored items; higher scores represent higher levels of prosocial motivation. In the present study, the Cronbach’s alpha coefficient for this scale was 0.863.

#### Subjective well-being

Subjective well-being was measured using the Index of Well-Being (IWB; Campbell et al., 1976), which has demonstrated good reliability and validity in previous research (Wang and Yang, 2021). The scale consists of two dimensions: the Index of General Affect (comprising the first eight items) and Life Satisfaction (the final single item). The consistency coefficient between the two dimensions was 0.55. Items B, D, E, and H are reverse-scored. The total score is calculated by adding the mean score of the General Affect dimension (weighted at 1.0) to the score of the Life Satisfaction dimension (weighted at 1.1). Responses are rated on a 7-point scale, with total scores ranging from 2.1 (least happy) to 14.7 (happiest); higher scores indicate higher levels of subjective well-being. In the present study, the Cronbach’s alpha for the General Affect dimension was 0.916.

#### Social desirability

Social desirability was measured using the shortened Chinese version of the Marlowe-Crowne Social Desirability Scale (MCSDS), which was revised and validated by Wei et al. (2015) and has demonstrated good reliability and validity in the Chinese context. The scale consists of 13 items with a dichotomous response format (Yes/No). Participants are asked to evaluate whether the description of each item matches their actual situation, with “No” scored as 0 and “Yes” scored as 1. Higher total scores indicate a stronger tendency toward social desirability. Among the items, three are positively phrased (Items 7, 8, and 11), such as “I am always willing to admit it when I make a mistake,” while the remaining 10 items are reverse-coded, such as “I sometimes like to gossip about others.” In the present study, the Cronbach’s alpha coefficient for this scale was 0.767.

#### Data analysis

Data analysis for this study was conducted using SPSS 26.0 alongside the PROCESS macro (Version 4.2, Model 8) developed by Hayes (2018). SPSS was utilized for sample descriptive statistics, bivariate correlation analysis, and regression assumption testing, while the PROCESS macro was employed to test the hypothesized moderated mediation model.

The analysis began with a descriptive overview of demographic variables to characterize the participant sample. To ensure the data were suitable for linear modeling, we tested the fundamental regression assumptions, including normality and multicollinearity. The Central Limit Theorem (CLT) suggests that in large samples (typically *N* > 30, and specifically in our sample of *N* = 1,084), the sampling distribution of the mean remains approximately normal, ensuring the robustness of parametric statistical tests (Ghasemi and Zahediasl, 2012). Additionally, all Variance Inflation Factor (VIF) values were below the threshold of 5, confirming that multicollinearity was not a concern (Hair et al., 2019). Crucially, to control for potential confounding, residence (urban or rural), social desirability, sex (male, female), political affiliation (CPC member, Non-CPC member), age, and grade level (Freshman, Sophomore, Junior, Senior, Graduate level, and above) were included as covariates throughout the entire analysis. Specifically, social desirability was conceptualized not merely as a mechanical statistical control, but as a critical confounder and a response style bias. Because self-reported prosociality in Chinese collectivist culture can be heavily influenced by the desire to conform to social norms (impression management), controlling for social desirability allows us to purify the prosocial behavior variable (Carlo and Randall, 2002). Other demographic variables were selected because previous studies have demonstrated their significant influence on prosocial behavior and subjective well-being (Hui et al., 2020; Yang and Wiepking, 2021). Categorical covariates (i.e., grade level and political affiliation) were dummy-coded prior to analysis. All continuous variables were standardized prior to the macro execution to facilitate the interpretation of results and reduce non-essential multicollinearity.

The core hypotheses were tested using Hayes (2018) PROCESS Model 8, which utilizes ordinary least squares (OLS) regression to estimate the conditional indirect pathways within a parallel multiple moderated mediation framework. In this model, prosocial behavior was the independent variable (X), subjective well-being was the dependent variable (Y), and prosocial motivation was the moderator (W). Crucially, rather than utilizing a total score, meaning in life was separated into its two distinct dimensions, search for meaning (M1) and presence of meaning (M2), which were entered simultaneously as parallel mediators. Consistent with Model 8 specification, the moderation effect of motivation was simultaneously modeled on both the pathway from behavior to both mediators (first stage) and the direct pathway from behavior to well-being. To evaluate the significance of the conditional indirect pathways, we generated 95% bias-corrected confidence intervals (CIs) based on 5,000 bootstrap resamples. A pathway was considered statistically significant if its 95% bootstrap confidence interval did not contain zero.

When the interaction between prosocial behavior and prosocial motivation yielded significance, a simple slope analysis was performed following the procedures of Aiken and West (1991). Specifically, the conditional effects of prosocial behavior were calculated and visualized at high and low levels of prosocial motivation, defined as one standard deviation above and below the sample mean (+1 SD and −1 SD), respectively. Finally, the overall index of moderated mediation was examined to determine whether the indirect pathway was significantly conditioned by the moderator.

## Results

Pearson correlation analysis was conducted to examine the relationships among the primary study variables (see Table 2). Results indicated that greater engagement in prosocial behavior was associated with higher levels of search for meaning (*r* = 0.33, *p* < 0.001), presence of meaning (*r* = 0.48, *p* < 0.001), and subjective well-being (*r* = 0.48, *p* < 0.001). Regarding the control variables, social desirability and age exhibited significant but low-magnitude associations with all primary research variables (*p* < 0.001).

**Table 2:** Correlations between the study variables.

Variables	Mean ± *SD*	1	2	3	4	5	6	7
1. Prosocial behavior	55.53 ± 13.85	1						
2. Search for meaning	20.39 ± 4.39	0.33^***^	1					
3. Presence of meaning	23.28 ± 6.28	0.48^***^	0.35^***^	1				
4. Subjective well-being	10.34 ± 2.36	0.48^***^	0.37^***^	0.65^***^	1			
5 Prosocial motivation	18.89 ± 3.36	0.45^***^	0.40^***^	0.46^***^	0.43^***^	1		
6. Social desirability	7.59 ± 3.15	0.19^***^	0.08^***^	0.29^***^	0.24^***^	0.27^***^	1	
7. Age	20.15 ± 1.82	0.38^***^	0.15^***^	0.28^***^	0.28^***^	0.27^***^	0.05	1

*p < 0.05, **p < 0.01, ***p < 0.001. SD, standard deviations.

For the equation focusing on search for meaning (M1), the overall model explained 22.40% of the total variance, *F*(13, 1,070) = 23.77, *p* < 0.001. After controlling for the covariates, prosocial behavior was significantly associated with higher search for meaning (*β* = 0.17, 95%CI [0.11, 0.24] *p* < 0.001). Crucially, prosocial motivation significantly moderated the relationship between prosocial behavior and search for meaning (*β* = 0.16, *p* < 0.001, 95%CI [0.11, 0.21]), explaining an additional 2.61% of the variance, *F*(1, 1,070) = 36.16, *p* < 0.001. Meanwhile, the equation with presence of meaning explained 36.74% of the total variance, *F*(13, 1,070) = 47.81, *p* < 0.001. Prosocial behavior was significantly associated with higher presence of meaning (*β* = 0.26, 95% CI [0.20, 0.31], *p* < 0.001), and prosocial motivation significantly moderated the relationship between prosocial behavior and presence of meaning (*β* = 0.16, 95% CI [0.11, 0.20], *p* < 0.001), explaining an additional 2.53% of the variance, *F*(1, 1,070) = 41.68, *p* < 0.001. These significant interaction terms confirmed that the first stage of both parallel mediation pathways was significantly moderated by prosocial motivation.

For the final equation with subjective well-being (Y) as the primary outcome, the variables collectively accounted for 48.96% of the variance, *F*(15, 1,068) = 68.66, *p* < 0.001. Both parallel mediators were significantly and positively associated with subjective well-being, with presence of meaning exhibiting a substantially stronger relationship (*β* = 0.45, 95% CI [0.40, 0.50], *p* < 0.001) compared to search for meaning (*β* = 0.10, 95% CI [0.05, 0.15], *p* < 0.001). Furthermore, the direct link between prosocial behavior and subjective well-being remained significant (*β* = 0.16, 95% CI [0.10, 0.21], *p* < 0.001). Additionally, prosocial motivation significantly moderated this direct relationship between prosocial behavior and subjective well-being (*β* = 0.10, 95% CI [0.05, 0.14], *p* < 0.001).

At the average level of the moderator, the parallel mediation analysis revealed that the indirect links between prosocial behavior and subjective well-being were statistically significant through both search for meaning (β_indirect_ = 0.02, 95% CI [0.01, 0.03]) and presence of meaning (β_indirect_ = 0.12, 95% CI [0.09, 0.15]). Crucially, the Index of Moderated Mediation was statistically significant for both parallel pathway, as evidenced by the 95% bootstrap confidence intervals excluding zero (Search pathway: Index = 0.016, SE = 0.01, 95% CI [0.01, 0.03]; Presence pathway: Index = 0.07, SE = 0.01, 95% CI [0.05, 0.10]).

**Figure 2:**
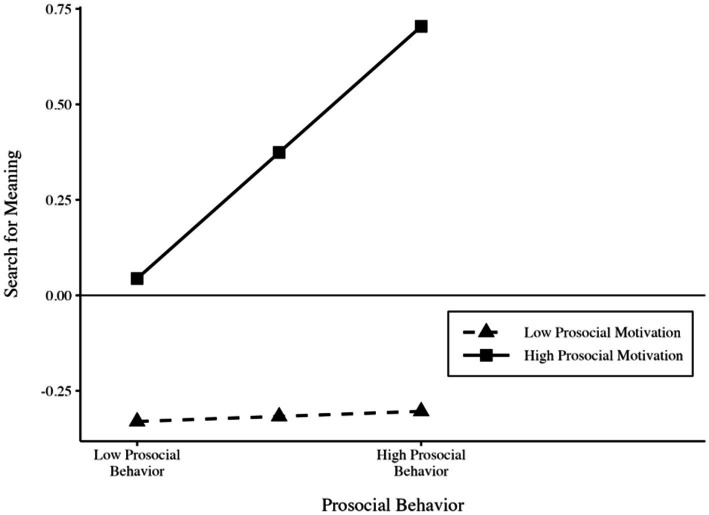
Simple slope analysis of the interaction between prosocial behavior and prosocial motivation on search for meaning.

**Figure 3:**
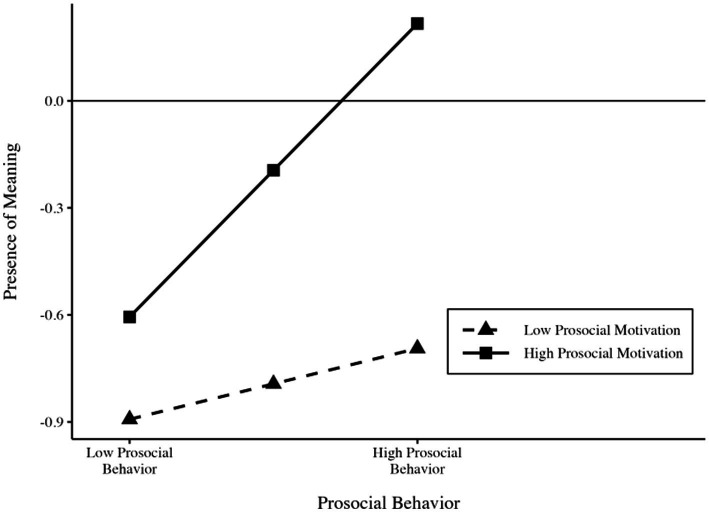
Simple slope analysis of the interaction between prosocial behavior and prosocial motivation on presence of meaning.

**Figure 4:**
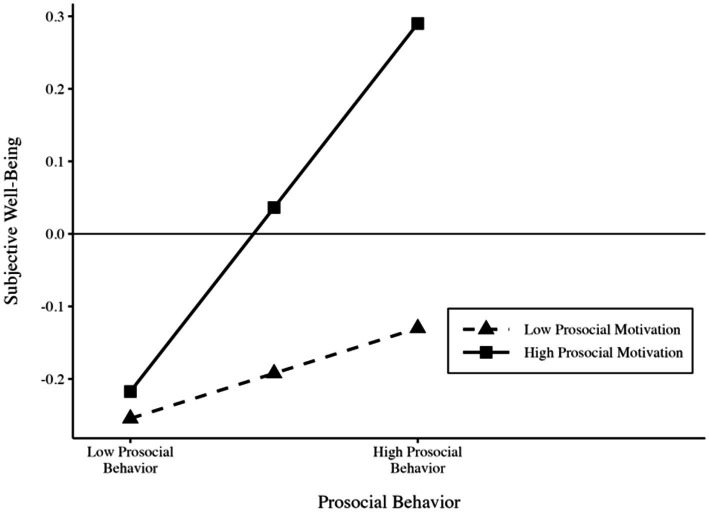
Simple slope analysis of the interaction between prosocial behavior and prosocial motivation on subjective well-being.

To decompose the specific conditional patterns across these interactions, a simple slope analysis was conducted by partitioning prosocial motivation into low (−1SD), moderate (0SD), and high (+1SD) levels. As illustrated in Figure 2, for students with low levels of prosocial motivation, the association between prosocial behavior and search for meaning was non-significant (*β* = 0.01, *t*(1071) = 0.30, *p* = 0.766, 95%CI [−0.08, 0.10]); however, for those with high levels of prosocial motivation, this positive relationship became strong and highly significant (*β* = 0.33, *p* < 0.001, 95% CI [0.25, 0.41]). In contrast, as illustrated in Figure 3, the positive link between prosocial behavior and presence of meaning remained statistically significant even under low prosocial motivation (*β* = 0.10, *p* = 0.014, 95% CI [0.02, 0.18]), and grew substantially stronger under high motivation conditions (*β* = 0.41, *p* < 0.001, 95% CI [0.34, 0.48]). Regarding the direct link to subjective well-being (Figure 4), the relationship was non-significant at low motivation (*β* = 0.06, *p* = 0.088, 95% CI [−0.01, 0.13]) but turned robustly positive and significant under high motivation (*β* = 0.25, *p* < 0.001, 95% CI [0.19, 0.32]). Finally, as summarized in Table 3, the conditional indirect effect size through the parallel pathways manifested an identical operational divergence: under the low motivation condition, the indirect link via search for meaning was non-significant, whereas the indirect link via presence of meaning remained consistently robust and significant.

**Table 3:** Conditional indirect effect size of standardized prosocial behavior on subjective well-being.

Mediator pathway	Prosocial motivation level (SD)	Indirect effect size	SE	95% CI
Prosocial Behavior → Search → SWB	Low (−1 SD)	0.00	0.006	[−0.01, 0.01]
	Moderate (0 SD)	0.02	0.006	[0.01, 0.03]
	High (+1 SD)	0.03	0.010	[0.01, 0.05]
Prosocial Behavior → Presence → SWB	Low (−1 SD)	0.05	0.020	[0.01, 0.08]
	Moderate (0 SD)	0.12	0.016	[0.09, 0.15]
	High (+1 SD)	0.19	0.021	[0.15, 0.23]

SWB, Subjective Well-Being. All variables were standardized prior to model estimation. Confidence intervals (CIs) that do not contain zero indicate statistical significance at the 0.05 level. SE, standard error; Search, search for meaning; Presence, presence of meaning.

## Discussion

The present study examined the complex interplay involving the mediating role of search for meaning and presence of meaning in the relationship between prosocial behavior and subjective well-being, as well as the moderating effect of prosocial motivation on this process among a sample of Chinese “Post-00s” college students. The results supported a moderated parallel mediation model. Frequent prosocial behavior was associated with higher subjective well-being, both directly and indirectly through the search for and presence of meaning. Crucially, this positive indirect pathway is contingent upon the level of prosocial motivation; the effect size of prosocial behavior on well-being is significantly amplified when individuals possess high prosocial motivation. A detailed discussion and analysis of these findings are provided below.

Specifically, when prosocial motivation was low, the search for meaning and direct pathways to well-being were non-significant, while the presence of meaning pathway remained robust. This can be interpreted through Self-Determination Theory (SDT; Ryan and Deci, 2017). When individuals engage in prosocial behaviors with a low internal desire, helping fails to spark the deep cognitive reflection required for search for meaning or the emotional resonance needed to boost subjective well-being directly. Nevertheless, the presence of meaning pathway remains significant. Even when the prosocial motivation is weak, prosocial behaviors often receive positive external feedback and social validation. This form of basic social interaction may sustain a baseline sense of meaning. This suggests that low-motivation prosociality acts as a “buffer” rather than a “catalyst”: it provides surface-level validation that maintains a sense of belonging, but fails to ignite the transformative cognitive processes necessary for deeper development. Therefore, the psychological benefits of prosocial behavior are conditional: when internal drive is low, prosociality is limited to satisfying the need for relatedness (maintaining presence), whereas a strong, internal desire to help is necessary to observe links to deeper cognitive growth (search) and overall well-being.

While not directly measured in the current data, this finding offers interesting insights when viewed against the cultural backdrop of contemporary Chinese youth. Previous literature suggests that younger generations may increasingly value psychological authenticity alongside traditional expectations (Sun and Ryder, 2016). For these individuals, engaging in prosocial behavior without a strong internal motivation represents an expenditure of personal resources without yielding significant psychological nourishment. In contrast, when prosocial behavior is driven by a high level of motivation, it fosters a strong sense of agency. Ultimately, the psychological benefits of helping are not automatic; they depend on the strength of motivation. This demonstrates that the internal intensity of the desire to help acts as a critical moderator, determining whether prosociality is weakly or strongly associated with genuine well-being.

### Theoretical and practical implications

The findings of this study offer several practical insights for mental health practitioners and higher education administrators in China.

First, our results highlight that while prosocial behavior can be a powerful driver of well-being, its association is strictly governed by the individual’s internal prosocial motivation. Therefore, universities should be cautious regarding “compulsory altruism” or “form-only” volunteerism (e.g., mandating volunteer hours for academic credits). Instead, institutions should focus on providing diverse prosocial opportunities that allow students to develop a stronger internal desire to help. When students engage in prosocial behaviors that they genuinely want to perform, they are more likely to report a sense of meaning and lasting happiness.

Second, the mediating role of both search for and presence of meaning suggests that psychological interventions for college students could benefit from incorporating prosocial therapy. By guiding students to engage in meaningful social contributions, counselors can help them achieve self-transcendence, thereby enhancing their sense of existential value and providing a buffer against psychological distress. For contemporary youth navigating a highly competitive academic and social environment, finding meaning through authentic connection may be a key pathway to mental resilience.

Moreover, this study provides important insights for research in the fields of prosocial behavior and subjective well-being, particularly by testing these associations within a non-Western student population.

First, this research refines the “doing good-feeling good” paradigm by establishing critical motivational boundary conditions. While previous literature often assumes a universal positive link between prosociality and well-being (Curry et al., 2018), our findings challenge this “one-size-fits-all” narrative. By demonstrating that the SWB benefits of altruism are neutralized under low prosocial motivation, we move the field beyond the simple “frequency” of helping and emphasize the strength of motivation required for a “warm glow.” This adds a layer of theoretical nuance, suggesting that for contemporary youth, the emotional rewards of helping are earned through volitional authenticity, not just behavioral repetition.

Second, the study extends Self-Determination Theory (SDT) by demonstrating how internal motivation differentially impacts distinct dimensions of meaning-making. By splitting meaning in life into search and presence, we provide empirical evidence that a strong desire to help is linked to active pursuit of meaning (Search), whereas the baseline realization of meaning (Presence) is slightly more resilient but still maximized by high motivation. This clarifies the precise cognitive and existential processes through which helping others is associated with well-being.

### Limitations and future directions

Despite the significant findings, several limitations of the current study must be acknowledged.

First, the use of a cross-sectional design limits our ability to draw firm causal inferences. Although our model is theoretically grounded, it is possible that individuals with higher subjective well-being are naturally more inclined to engage in prosocial behavior. Future research should employ longitudinal designs or experimental interventions (e.g., random assignment to prosocial tasks with different motivational primes) to better establish the causal directionality of these relationships.

Second, the sample was recruited through convenience sampling from four universities in a single city in Yunnan Province. Therefore, the representativeness of the sample is limited, and caution must be exercised when generalizing these findings to the broader population of Chinese youth. Future studies should utilize larger, nationally representative samples to validate these findings across different regions.

Third, although we controlled for social desirability, all variables were assessed via self-report measures. Given the high value placed on altruism in Chinese culture, participants might still have over-reported their prosocial behaviors due to “face” (Mianzi) concerns. Future studies could benefit from multi-informant approaches (e.g., peer or teacher ratings) or behavioral observations to ensure greater data objectivity.

Finally, while we contextualized our findings within the cultural backdrop of the Chinese “Post-00s” generation navigating individualism and collectivism, these broader cultural constructs (e.g., psychological authenticity, specific cultural values, or institutional volunteering requirements) were not directly measured in this study. The current data solely support a statistical moderation model based on self-reported motivation and behavior among a convenience sample in Yunnan Province. Therefore, future research should explicitly measure these external environmental and cultural factors in larger, nationally representative samples to directly test the broader cultural mechanisms influencing students’ prosocial behavior.

## Conclusion

In conclusion, this study demonstrates that meaning in life serves as a vital statistical link between prosocial behavior and subjective well-being, yet this entire process is critically contingent upon the level of prosocial motivation. Viewed within the context of a generation navigating the tension between traditional collectivist norms and personal goals, the positive association between helping and well-being is primarily observed when individuals possess a strong, internalized desire to benefit others. These findings indicate that to sustain the statistical alignment between prosociality and subjective well-being among contemporary youth, focus should be placed on fostering a genuine, strong motivation to help, rather than merely encouraging behavioral frequency.

## Data Availability

The raw data supporting the conclusions of this article will be made available by the authors, without undue reservation.
